# Supplementary material for: Chalcone Derivative Induces Flagellar Disruption and Autophagic Phenotype in Phytomonas serpens In Vitro
Source: Pathogens. 2023 Mar 7;12(3):423. doi: 10.3390/pathogens12030423 (PMC10051746; doi:10.3390/pathogens12030423)
Supplement: Supplementary file 1 [file pathogens-12-00423-s001.zip › pathogens-2228305-supplementary.pdf]

General procedure for the synthesis and identification of the derivative NaF

The derivative NaF was synthesized by Claisen–Schmidt condensation using a solution of 2.2 g of NaOH in 32 mL of water/ethanol (2:1), followed by 5.2 g of acetophenone derivative and 4.6 g of the corresponding aldehyde. The reaction medium was kept in an ice bath for 5 minutes, then removed and retained at room temperature for 3 hours. After that, the mixture was stored in the freezer for 12 hours and then filtered by suction.

A gas chromatograph equipped with a mass spectrometer (Shimadzu GC-MS-QP2010 – Kyoto, Japan) was used for mass spectrum determinations using a Rtx®-5MS Restek fused silica capillary column (5% -diphenyl-95%-dimethyl polysiloxane) of 30 m, 0.25 mm internal diameter (di), 0.25 µm film thickness, in a constant helium flow (99.999%) with a rate of 1.2 mL min^{-1}. The volume of injection used was 1 µL (10 mg mL^{-1}), with a split ratio of 1:30. The oven temperature was programmed from 80 ˚C (isotherm for 1.0 min), with an increase of 6 ˚C/min, ending with a 10 min isotherm at 300 ˚C.The molecules were ionized by electrons (EI) with energy of 70 eV and the fragments analyzed by a quadrupole analyzer programmed to filter fragments ions from *m/z* 40 to 600 Da and detected by an electron multiplier. The data processing was performed by the GC/MS software. HRMS analyses were recorded on a Bruker microTOF-Q II model device with "time off flight" analyzer and "Electrospray Ionization" source operating in positive mode (ESI^+).

Nuclear Magnetic resonance (NMR) spectra were recorded using a Varian Inova Spectrometer (500 MHz for ^1H and 125 MHz for ^{13}C NMR) in $CDCl_3$.All spectra were recorded at 25 °C and coupling constants (*J* values) are given in Hz. The chemical shifts are reported in parts per million (ppm) downfield from tetramethylsilane (TMS), which was used as the internal standard.

(*E*)-1-phenyl-3-α-naphthylprop-2-en-1-one (C-5)

Dark yelow solid (93%); m.p. 79-81 °C (Lit. - °C); ^1H NMR (600 MHz, $CDCl_3$) δ 8.72 (d, *J* = 15.4 Hz, 1H), 8.30 (d, *J* = 8.5 Hz, 1H), 8.13 (m, 2H), 7.96 (m, 3H), 7.68 (d, *J* = 15.4 Hz, 1H), 7.60 (m, 6H); ^{13}C NMR (150 MHz, $CDCl_3$) δ 190.5 (C), 141.7 (CH), 138.2 (C), 133.7 (C), 133.6 (C), 133.6 (CH), 130.7 (C), 129.0 (CH), 128.7 (CH), 128.5 (CH), 128.4 (CH), 126.9 (CH), 126.3 (CH), 125.6 (CH), 125.4 (CH), 124.6 (CH), 123.4 (CH); HR-MS *m/z* [M+1] 259.1129; EIMS: *m/z* 258 $[M]^{+\cdot}$, 257, 230, 181, 153, 147, 105, 77; FT-IR (KBr, cm^{-1}): ν_{Max} 3046, 1661, 1602.

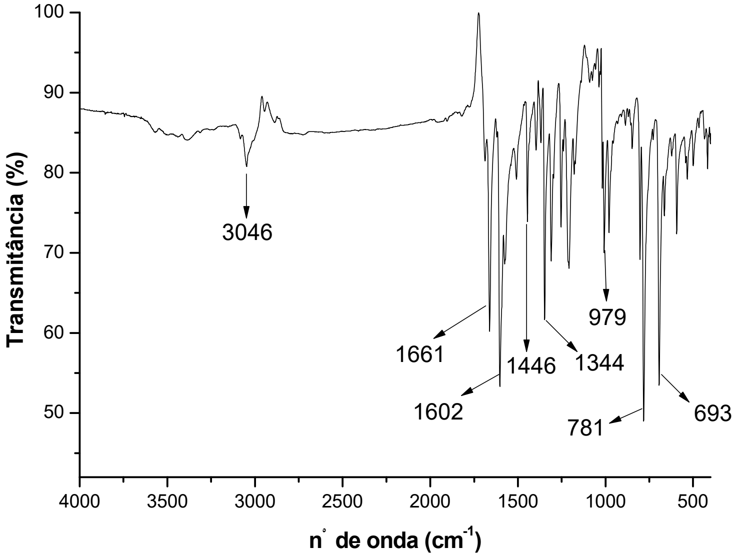

Figure S1. Infrared absorption spectrum of the derivative **NaF.**

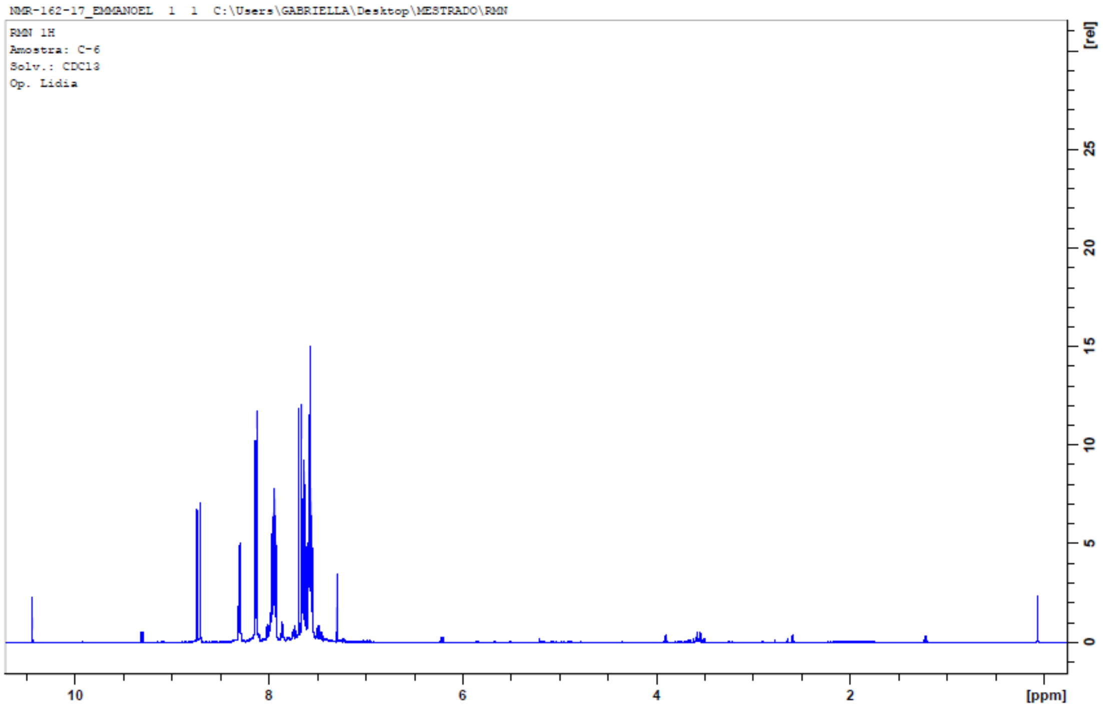

Figure S2. ^{1}H-NMR (500 MHz, $CDCl_3$) spectrum of the derivative **NaF.**

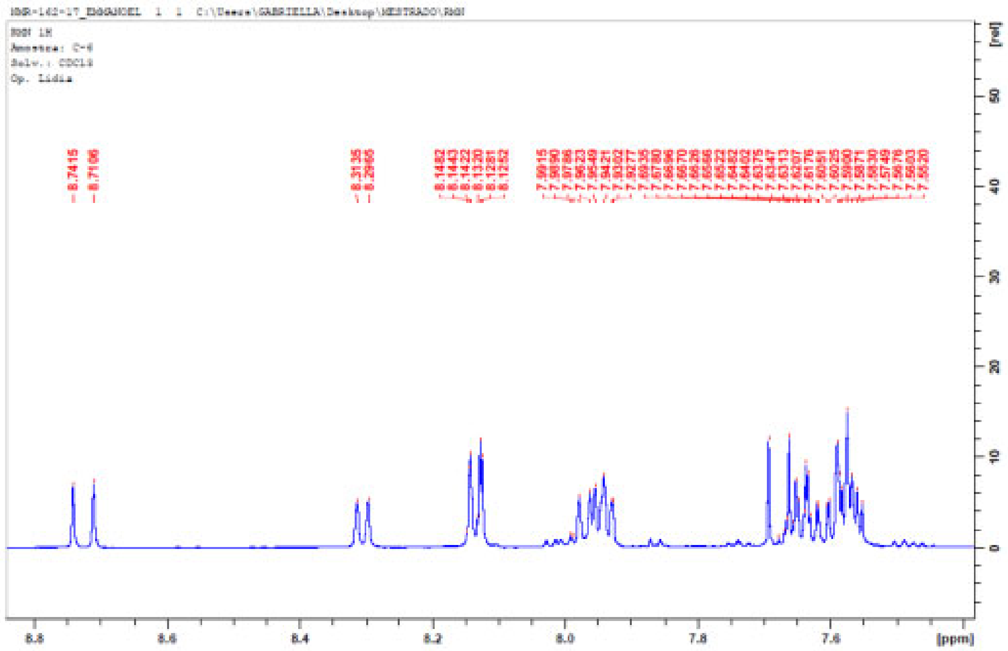

Figure S3. ^{1}H-NMR (500 MHz, $CDCl_3$) expansion spectrum of the derivative **NaF.**

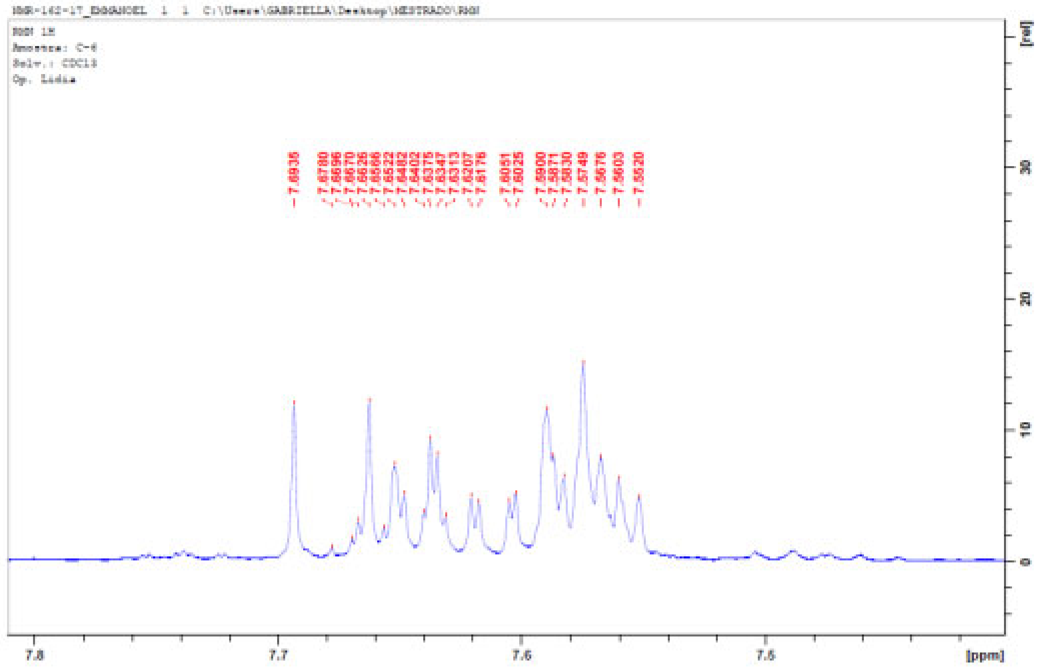

Figure S4. ^{1}H-NMR (500 MHz, $CDCl_3$) expansion spectrum of the derivative **NaF.**

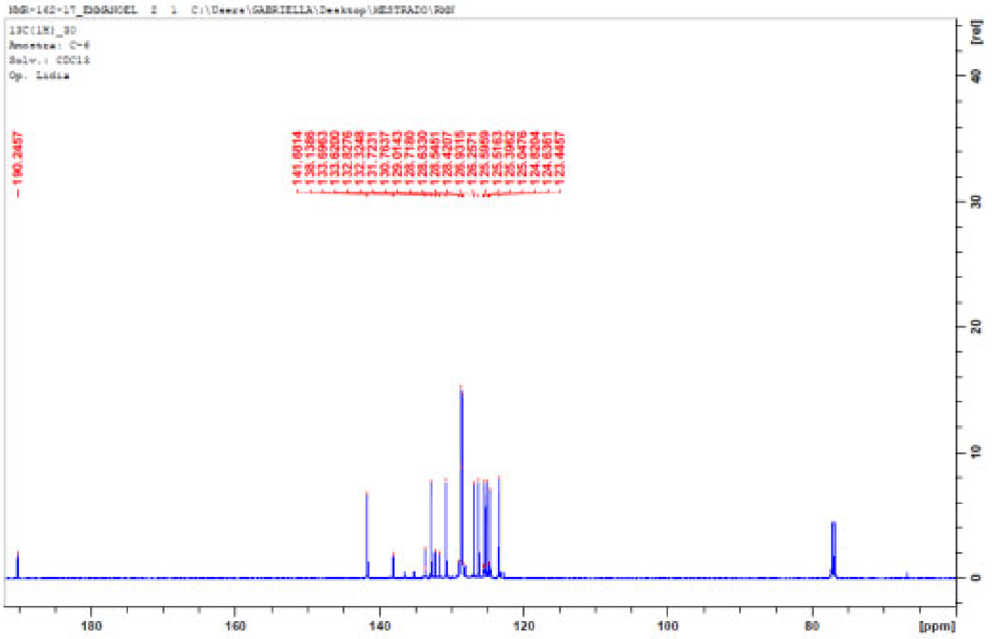

Figure S5. ^{13}C-NMR (125 MHz, $CDCl_3$) spectrum of the derivative **NaF**.

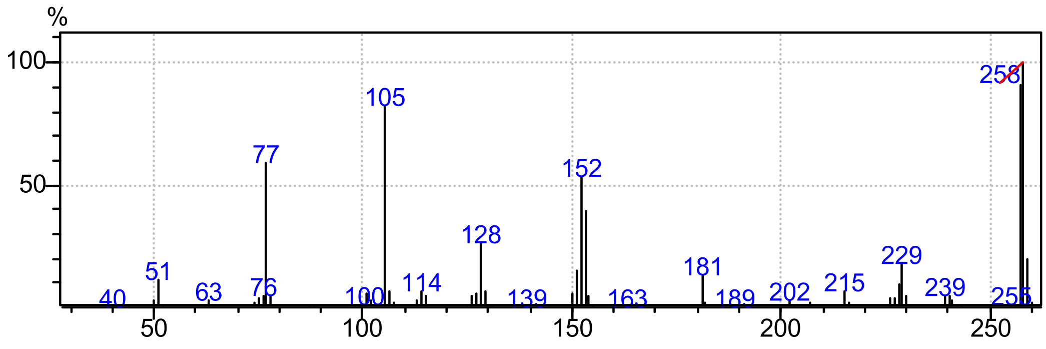

Figure S6. Mass spectrum of the derivative **NaF**.

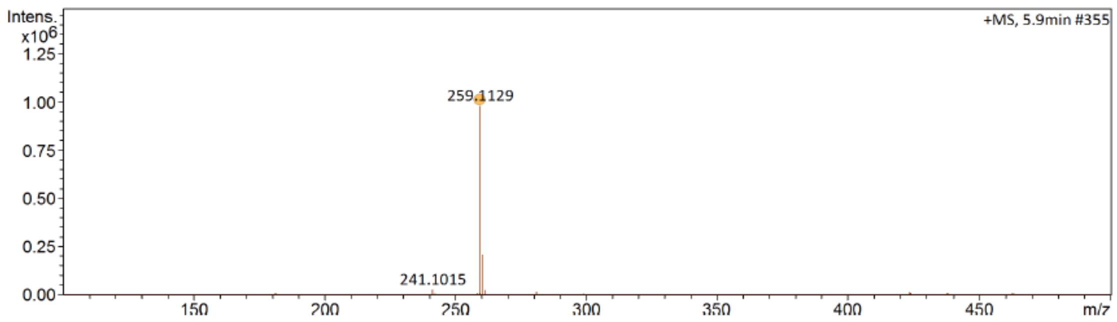

Figure S7. High resolution mass spectrum of the derivative **NaF**.
